# Theoretical prediction of a charge-transfer phase transition

**DOI:** 10.1038/s41598-017-18213-0

**Published:** 2018-01-11

**Authors:** Hiroko Tokoro, Asuka Namai, Marie Yoshikiyo, Rei Fujiwara, Kouji Chiba, Shin-ichi Ohkoshi

**Affiliations:** 1 Department of Chemistry, School of Science, The University of Tokyo, 7-3-1 Hongo, Bunkyo-ku, Tokyo 113-0033 Japan; 2 Division of Materials Science, Faculty of Pure and Applied Sciences, University of Tsukuba, 1-1-1 Tennodai, Tsukuba, Ibaraki 305-8573 Japan; 3 Material Science Div., MOLSIS Inc., Tokyo Daia Bldg., 1-28-38 Shinkawa, Chuo-ku, Tokyo 104-0033 Japan; 4 Cryogenic Research Center, The University of Tokyo, 2-11-16 Yayoi, Bunkyo-ku, Tokyo 113-0032 Japan

## Abstract

Phase transition materials are attractive from the viewpoints of basic science as well as practical applications. For example, optical phase transition materials are used for optical recording media. If a phase transition in condensed matter could be predicted or designed prior to synthesizing, the development of phase transition materials will be accelerated. Herein we show a logical strategy for designing a phase transition accompanying a thermal hysteresis loop. Combining first-principles phonon mode calculations and statistical thermodynamic calculations considering cooperative interaction predicts a charge-transfer phase transition between the A–B and A^+^–B^−^ phases. As an example, we demonstrate the charge-transfer phase transition on rubidium manganese hexacyanoferrate. The predicted phase transition temperature and the thermal hysteresis loop agree well with the experimental results. This approach will contribute to the rapid development of yet undiscovered phase transition materials.

## Introduction

Phase transition phenomena such as spin-crossover, charge-transfer, metal–insulator, and crystal–amorphous transitions have been aggressively studied^1–15^. Phase transition materials are attractive from the viewpoint of practical applications because external stimuli such as light, pressure, or an electric field can switch the physical properties of the material^16–39^. For example, crystal–amorphous phase transition materials are used in optical memories and resistance random access memories^1,2^. Spin-crossover complexes have been studied as color switching materials^6–8,16–20^, while lambda-trititanium-pentoxide has potential in optical memory devices and heat storage applications^28,35^. Cyanide-bridged bimetallic assemblies exhibit charge-transfer phase transitions^10–12,21–25^. One cyanide-bridged bimetallic assembly, rubidium manganese hexacyanoferrate, shows a charge-transfer phase transition, which can be induced by light, pressure, or an electric field^39–44^.

However, questions remain in phase transition materials research. Can phase transition materials be designed? Can switching materials originating from bistability be predicted? Herein we consider a strategy to predict phase transitions. Estimations of thermodynamic parameters, such as enthalpy, entropy, and Gibbs free energy, are necessary to predict phase transitions. Additionally, the cooperative interaction inside the crystal at the phase transition must be evaluated to predict a thermal hysteresis loop. In this study, we predict a charge-transfer phase transition on rubidium manganese hexacyanoferrate as a demonstration system using a combination method between first-principles calculations and statistical thermodynamic calculations. To confirm our prediction, we synthesize the material and evaluate the validity of our prediction.

**Figure 1:**
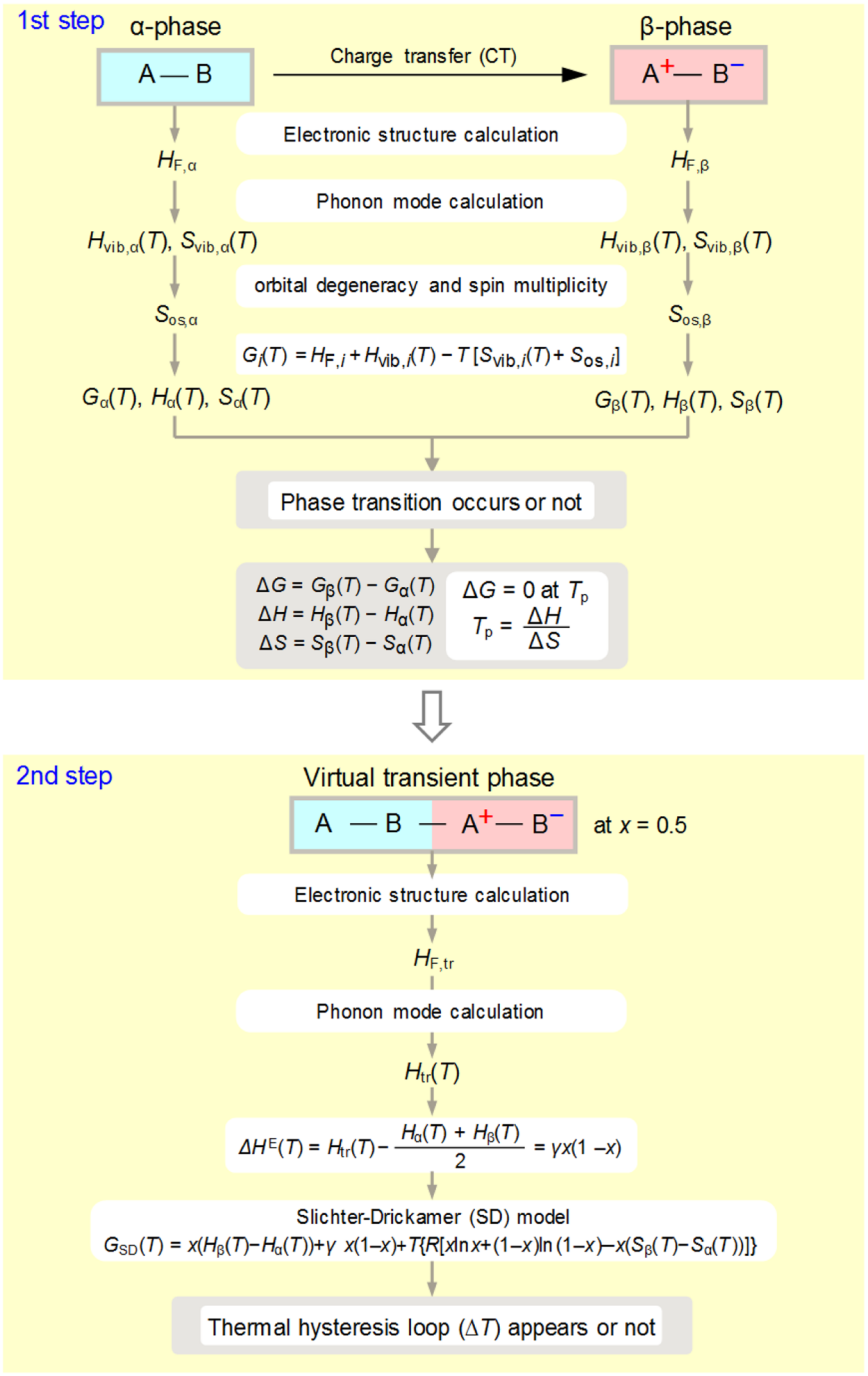
Strategy to predict a phase transition and thermal hysteresis loop. Flowchart showing the strategy to predict a phase transition (upper). Formation energies and thermodynamic parameters of two bistable phases and the transition temperature (*T*_p_) are obtained. Lower flowchart shows the strategy to predict a thermal hysteresis loop. Thermodynamic parameters of the virtual transient phase, excess enthalpy (∆*H* ^E^), and interaction parameter (*γ*) are obtained. Then the thermal hysteresis loop (∆*T*) is estimated by the SD model calculation.

As a first step, we considered a flowchart of a logical strategy to predict the charge-transfer phase transition between the A–B phase (α-phase) and its valence isomer where one electron transfers from A to B, *i.e*., A^+^–B^−^ phase (β-phase) (Fig. 1). On the basis of first-principles calculation of the electronic structure and phonon modes, the crossover temperature between the Gibbs energies for the α-phase (*G*_α_(*T*)) and β-phase (*G*_β_(*T*)) corresponding to the phase transition temperature (*T*_p_) is predicted. As a second step, to determine whether a system has a thermal hysteresis, the statistical thermodynamic Slichter-Drickamer (SD) model^45^, which is one of the mean-field regular solution models, is adopted. In the SD model, the excess enthalpy, Δ*H* ^E^, is expressed by *γx*(1− *x*), where *γ* is the interaction parameter due to the interaction between the A–B and A^+^–B^−^ units, and *x* is the fraction of the A^+^–B^−^ unit. To evaluate *γ*, we need to consider the virtual transient phase (tr-phase), such as the A–B–A^+^–B^–^ phase. From the results of SD model calculations, whether a thermal hysteresis loop appears or not can be determined. The detail explanation of the strategy mentioned above are described in the Methods section.

In the case of rubidium manganese hexacyanoferrate, the α- and β-phases correspond to the Fe^II^(*S* = 0)–CN–Mn^III^(*S* = 2) phase (Fe^II^–Mn^III^ phase) and the Fe^III^(*S* = 1/2)–CN–Mn^II^(*S* = 5/2) phase (Fe^III^–Mn^II^ phase), respectively. Hereafter, we try to predict the charge-transfer phase transition between the Fe^II^–Mn^III^ and Fe^III^–Mn^II^ phases.

## Results and Discussion

### First-principles electronic structure calculations of the Fe^II^–Mn^III^ and Fe^III^–Mn^II^ phases

To calculate the formation enthalpies of the Fe^II^–Mn^III^ and Fe^III^–Mn^II^ phases at zero kelvin, ${H}_{\text{F,}{\text{Fe}}^{\text{II}}{\text{Mn}}^{\text{III}}}$ and ${H}_{\text{F,}{\text{Fe}}^{\text{II}\text{I}}{\text{Mn}}^{\text{II}}}$, first-principles electronic structure calculations were performed using the Vienna *ab initio* Simulation Package (VASP). We adopted a screened Coulomb hybrid functional calculation of the Heyd–Scuseria–Ernzerhof (HSE06) hybrid functional as the calculation method (see Methods). HSE06 was selected because the formation enthalpies of the Fe^II^–Mn^III^ and Fe^III^–Mn^II^ phases must be compared precisely. The hybrid functional calculation is suitable because it calculates the formation enthalpy with a higher accuracy and does not require tuning parameters (such as *U*−*J* in GGA + U). The obtained values of ${H}_{\text{F,}{\text{Fe}}^{\text{II}}{\text{Mn}}^{\text{III}}}$ and ${H}_{\text{F,}{\text{Fe}}^{\text{II}\text{I}}{\text{Mn}}^{\text{II}}}$ are −14476.69 kJ mol^−1^ and −14454.33 kJ mol^−1^, respectively.

### First-principles phonon mode calculations of the Fe^II^–Mn^III^ and Fe^III^–Mn^II^ phases

**Figure 2:**
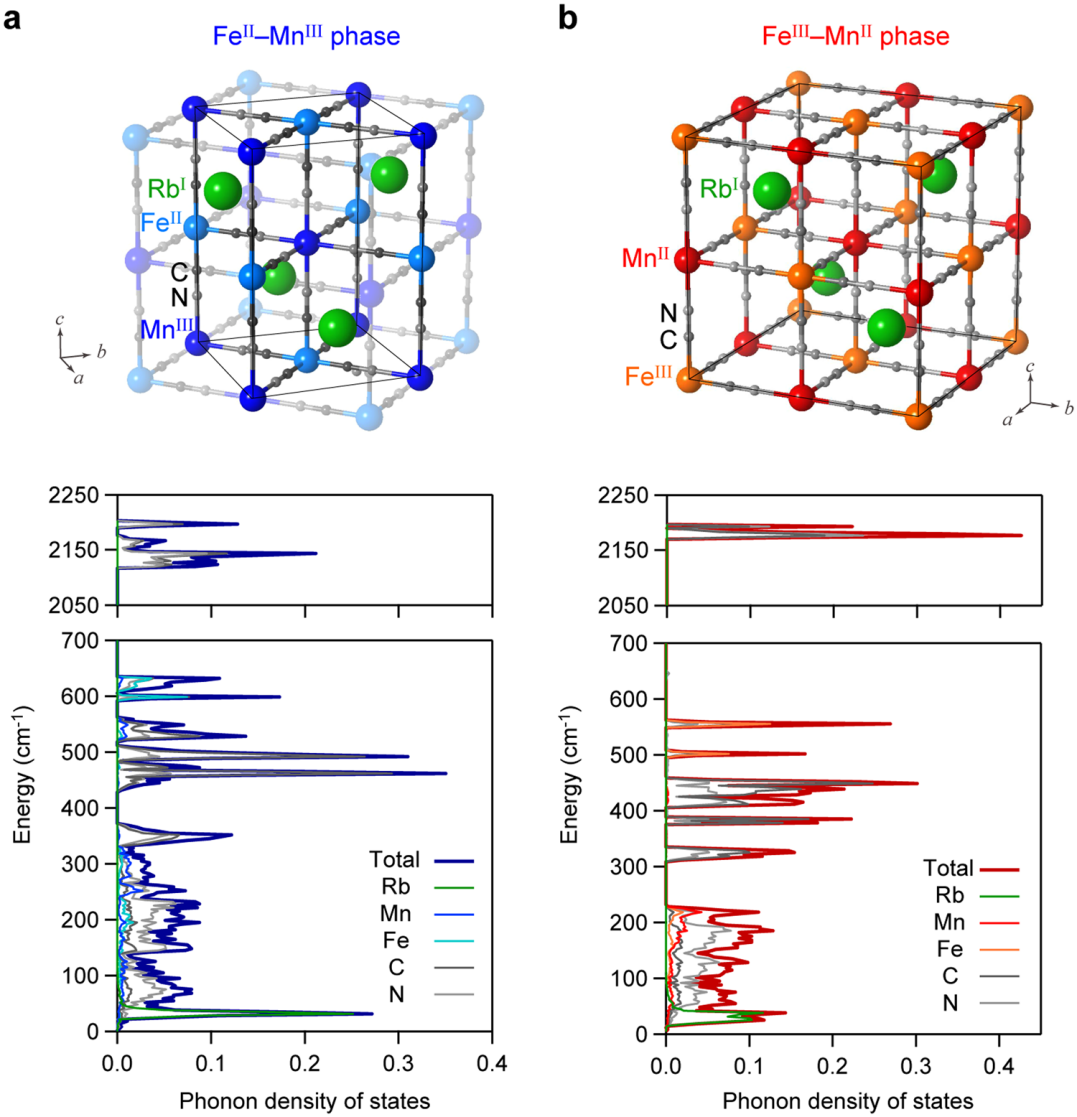
Crystal structures and phonon density of states. (**a**) Upper figure shows the initial crystal structure of the Fe^II^–Mn^III^ phase used in the first-principles calculations. Solid and semitransparent structures indicate the primitive cell and the unit cell, respectively. Green, blue, light blue, gray, and light gray balls represent Rb, Mn, Fe, C, and N atoms, respectively. Lower figure shows the phonon density of states calculated by first-principles phonon mode calculations. Dark blue line shows the total phonon density of states. Green, blue, light blue, gray, and light gray lines indicate the partial phonon density of states for Rb, Mn, Fe, C, and N, respectively. (**b**) Upper figure shows the initial crystal structure of the Fe^III^–Mn^II^ phase used for the first-principles calculations. Green, red, orange, gray, and light gray balls represent Rb, Mn, Fe, C, and N atoms, respectively. Lower figure shows the phonon density of states calculated by first-principles phonon mode calculations. Dark red line shows the total phonon density of states. Green, red, orange, gray, and light gray lines indicate the partial phonon density of states for Rb, Mn, Fe, C, and N atoms, respectively.

**Figure 3:**
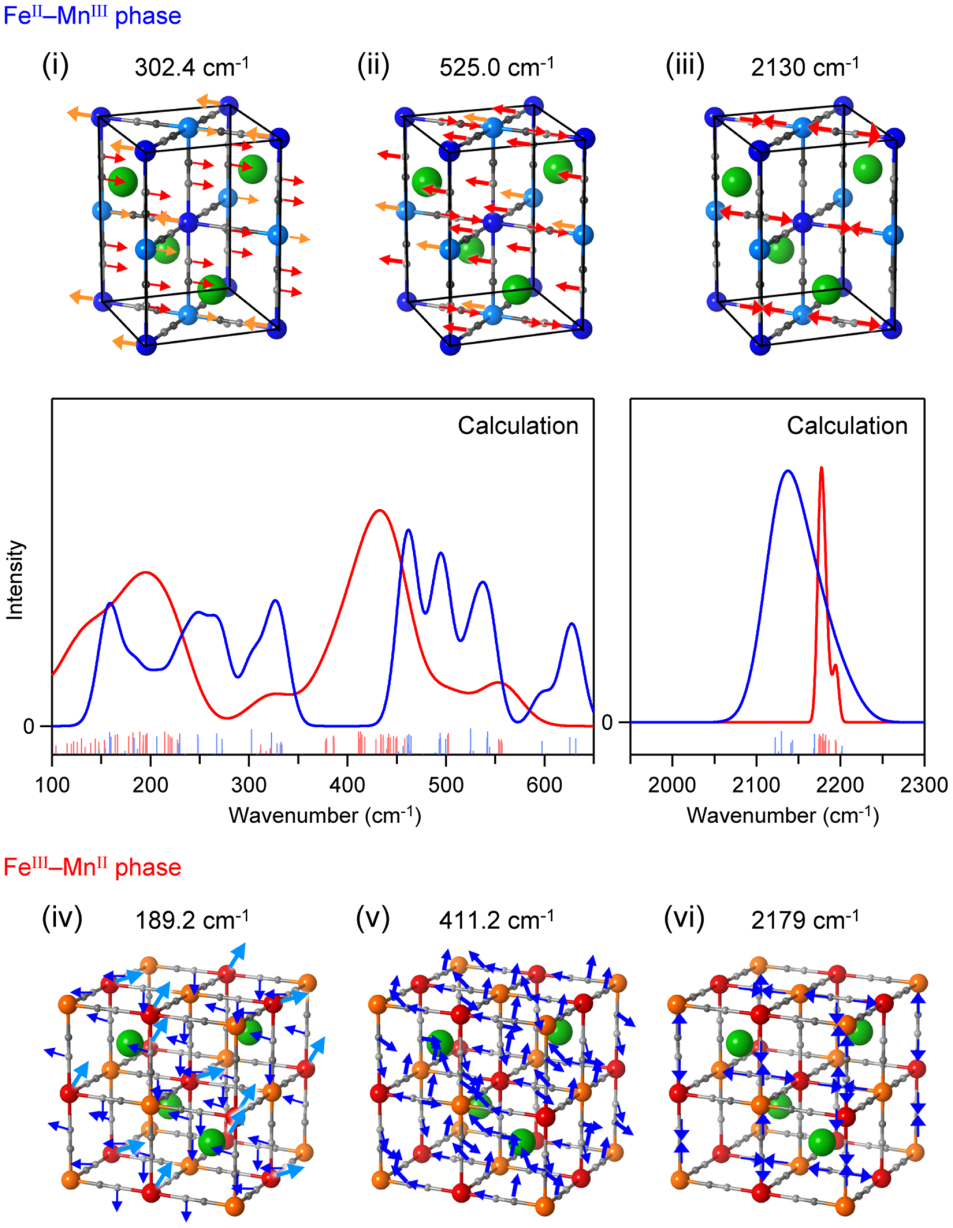
Calculated IR spectra and atomic movements of the phonon modes. Middle part shows the calculated IR spectra of the Fe^II^–Mn^III^ and Fe^III^–Mn^II^ phases shown with blue and red lines, respectively. Sticks indicate the positions and intensities of the IR active phonon modes for the Fe^II^–Mn^III^ (blue) and Fe^III^–Mn^II^ (red) phases. Upper part of the figure shows the atomic movements of the phonon modes of the Fe^II^–Mn^III^ phase at (i) 302.4 cm^−1^, (ii) 525.0 cm^−1^, and (iii) 2130 cm^−1^. Lower part shows the atomic movements of the phonon modes of the Fe^III^–Mn^II^ phase at (iv) 189.2 cm^−1^, (v) 411.2 cm^−1^, and (vi) 2179 cm^−1^.

The phonon modes of the Fe^II^–Mn^III^ phase with a tetragonal structure in the $I\bar{\text{4}}m\text{2}$ space group (Fig. 2a (upper panel) and Table S1) were calculated by first-principles calculations using the Phonon code with GGA + U/PBE. The Fe^II^–Mn^III^ phase has 29 optical phonon modes and 3 acoustic phonon modes. Figure 2a (lower panel) shows the calculated phonon density of states of the Fe^II^–Mn^III^ phase. Furthermore, the transition probabilities of the optical phonon modes were calculated. From the obtained transition probabilities, the IR active optical spectra were determined (Fig. 3, blue lines). In the spectra, the phonon modes due to the symmetric bending and asymmetric bending modes of Fe–C≡N–Mn appear in the region of 100–650 cm^−1^, while the phonon modes due to the stretching mode of C≡N appear in the region of 2050–2250 cm^−1^ (Fig. 3(i)–(iii)). As representative examples, Movie S1 shows the atomic movements of the phonon modes at 302.4 cm^−1^, 525.0 cm^−1^, and 2130 cm^−1^, respectively.

The phonon modes of the Fe^III^–Mn^II^ phase with a cubic structure (space group, $F\bar{\text{4}}\text{3}m$) were calculated (Fig. 2b upper and Table S2). The Fe^III^–Mn^II^ phase has 16 optical phonon modes and 3 acoustic phonon modes. Figure 2b (lower panel) shows the calculated phonon density of states of the Fe^III^–Mn^II^ phase. The IR active optical spectrum was calculated from the transition probabilities (Fig. 3, red lines). In the spectra, the phonon modes corresponding to the symmetric bending and asymmetric bending modes of Fe–C≡N–Mn appear in the region of 100–650 cm^−1^, while one peak appears due to the stretching mode of C≡N in the region of 2150–2200 cm^−1^ (Fig. 3(iv)–(vi)). As representative examples, Movie S2 shows the atomic movements of the phonon modes at 189.2 cm^−1^, 411.2 cm^−1^, and 2179 cm^−1^, respectively. From the results of the phonon mode calculations for the Fe^II^–Mn^III^ and Fe^III^–Mn^II^ phases, the vibrational enthalpy *H*_vib_(*T*) and vibrational entropy *S*_vib_(*T*) curves are obtained.

### Prediction of the phase transition and evaluation of the phase transition temperature

**Figure 4:**
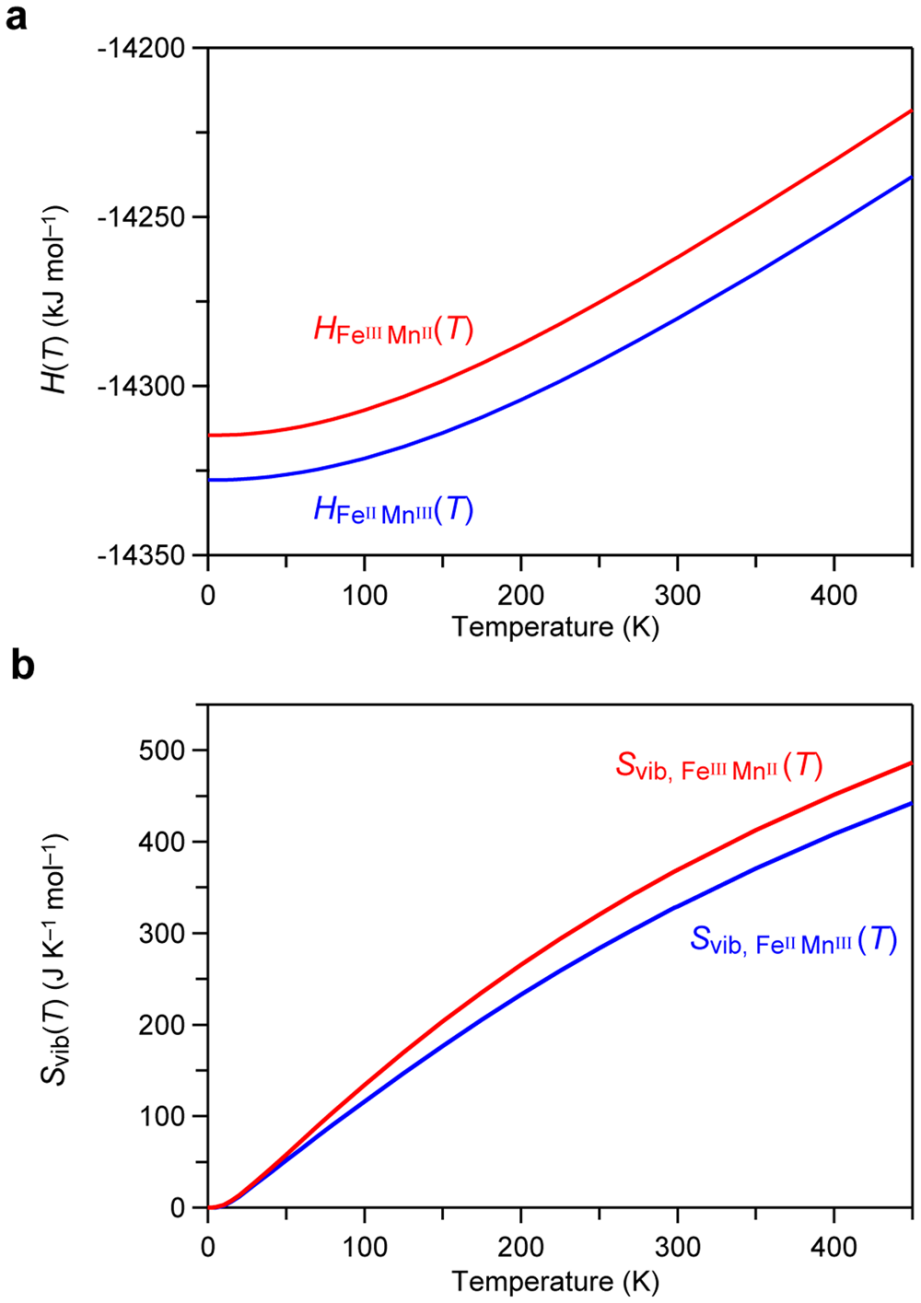
Thermodynamic parameters obtained by first-principles phonon mode calculations. Calculated temperature dependence of (**a**) enthalpy *H*(*T*), and (**b**) vibrational entropy *S*_vib_(*T*) for the Fe^II^–Mn^III^ phase (blue lines) and Fe^III^–Mn^II^ phase (red lines).

**Figure 5:**
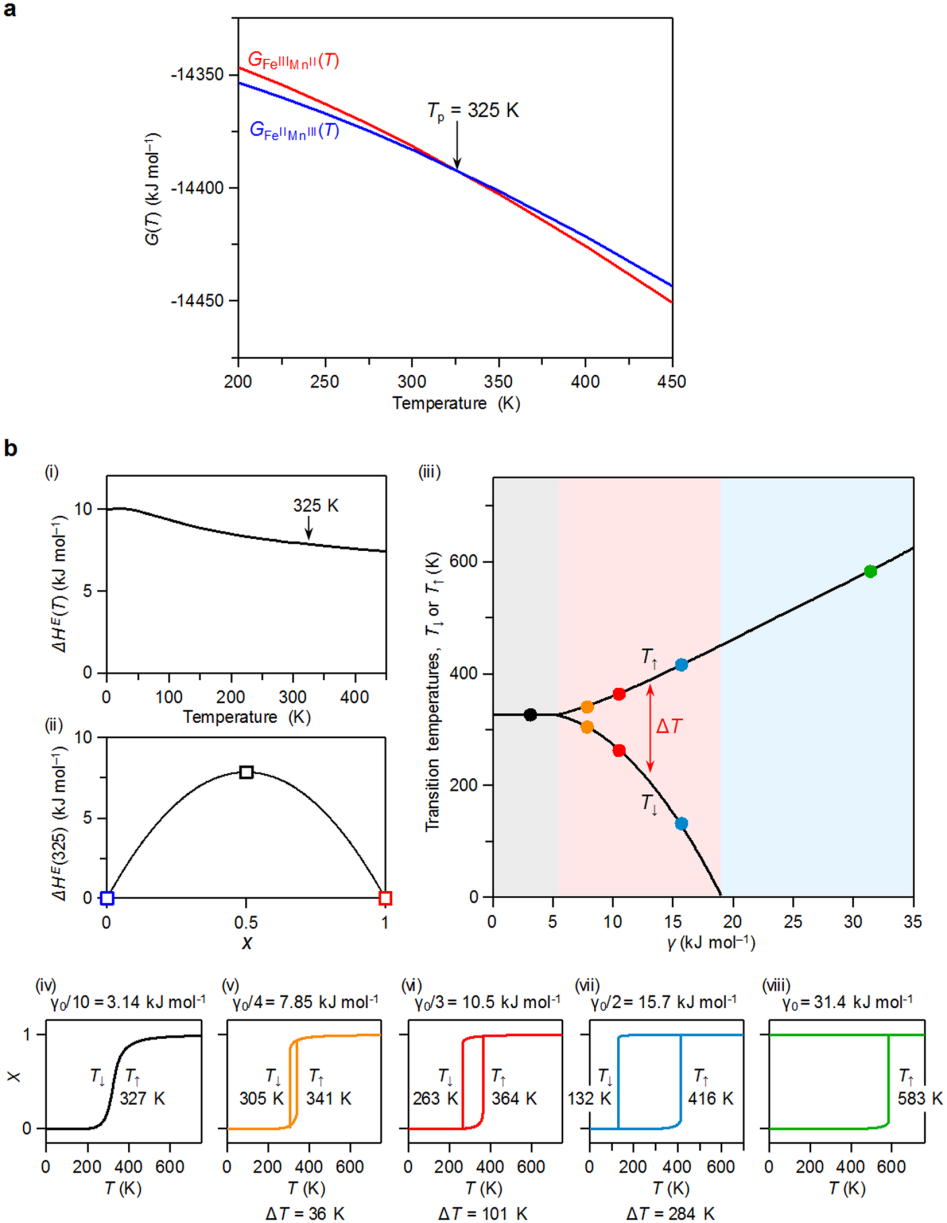
Prediction of the phase transition and thermal hysteresis loop. (**a**) Temperature dependence of Gibbs free energy *G*(*T*) obtained by first-principles phonon mode calculations for the Fe^II^–Mn^III^ phase (blue line) and Fe^III^–Mn^II^ phase (red line). ${G}_{{\text{Fe}}^{\text{II}}{\text{Mn}}^{\text{III}}}$(*T*) and ${G}_{{\text{Fe}}^{\text{III}}{\text{Mn}}^{\text{II}}}$(*T*) curves show a crossover at 325 K, which is the phase transition temperature (*T*_p_). (**b**) (i) Δ*H* ^E^(*T*) vs. *T* curve. Δ*H* ^E^(*T*) is obtained as ${\rm{\Delta }}{H}^{E}(T)$ $(={H}_{{\rm{tr}}}(T)-({H}_{{{\rm{Fe}}}^{{\rm{II}}}{{\rm{Mn}}}^{{\rm{III}}}}(T)+{H}_{{{\rm{Fe}}}^{{\rm{III}}}{{\rm{Mn}}}^{{\rm{II}}}}(T))/2)$ in the regular solution model. (ii) Diagram showing the calculated excess enthalpies (Δ*H* ^E^) of Fe^II^–Mn^III^ phase (blue square), Fe^III^–Mn^II^ phase (red square), and tr-phase (black square). (iii) Calculated *T*_↑_ and *T*_↓_ vs. *γ* plot. Black, orange, red, blue, and green filled circles correspond to the calculated *T*_↑_ and *T*_↓_ with *γ* values of *γ*_0_/10, *γ*_0_/4, *γ*_0_/3, *γ*_0_/2, and *γ*_0_, respectively. Calculated *x* vs. *T* curves based on the SD model using the thermodynamic parameters of Δ*H* = 18.43 kJ mol^−1^, Δ*S* = 56.44 J K^−1^ mol^−1^, and *γ* values of (iv) *γ*_0_/10, (v) *γ*_0_/4, (vi) *γ*_0_/3, (vii) *γ*_0_/2, and (viii) *γ*_0_.

Based on the first-principles electronic structure calculations and phonon mode calculations, we considered whether the charge-transfer induced phase transition occurs in rubidium manganese hexacyanoferrate. From the sum of the formation enthalpy (*H*_F_) and the vibrational enthalpy (*H*_vib_(*T*)), the temperature dependence of the enthalpy (*H*(*T*)) of each phase was obtained (Fig. 4a). On the other hand, the entropy (*S*(*T*)) of each phase was obtained as the sum of *S*_vib_(*T*) (Fig. 4b) and the contribution from the orbital degeneracy and the spin multiplicity (*S*_os_). The *S*_os_ value for the Fe^II^–Mn^III^ phase is *R*ln5 due to Fe^II^(^1^ *A*_1g_) and Mn^III^(^5^ *B*_1g_), while that for Fe^III^–Mn^II^ phase is *R*ln36 due to Fe^III^(^2^ *T*_2g_) and Mn^II^(^6^ *A*_1g_) (Supplementary Information). Using these thermodynamic parameters, the Gibbs free energies of the Fe^II^–Mn^III^ phase and the Fe^III^–Mn^II^ phase were evaluated. As shown in Fig. 5a, the ${G}_{{\text{Fe}}^{\text{II}}{\text{Mn}}^{\text{III}}}$(*T*) and ${G}_{{\text{Fe}}^{\text{III}}{\text{Mn}}^{\text{II}}}$(*T*) curves show a crossover at a specific temperature. This calculation predicts that the present system may show a charge-transfer phase transition at *T*_p_ = 325 K. At *T*_p_, transition enthalpy and transition entropy are ∆*H* = 18.43 kJ mol^−1^ and ∆S = 56.44 J K^−1^ mol^−1^, respectively. *T*he Fe^II^–Mn^III^ phase is stable below *T*_p_, while the Fe^III^–Mn^II^ phase becomes stable above *T*_p_.

### Evaluation of a thermal hysteresis loop in the phase transition

As a second step, we considered whether a thermal hysteresis loop appears in the present system. The *x* value of Δ*H* ^E^(*T*) = *γx*(1 − *x*) corresponds to the fraction of the Fe^III^–Mn^II^ phase, and *γ* is an interaction parameter between the Fe^II^–Mn^III^ and Fe^III^–Mn^II^ units. To obtain the *γ* value, we need to consider an intermediate phase between the Fe^II^–Mn^III^ and Fe^III^–Mn^II^ phases. Here, we set a virtual transient phase of (A–B layer)-by-(A^+^–B^−^ layer) structure (i.e., (Fe^II^–Mn^III^ layer)-by-(Fe^III^–Mn^II^ layer) structure) (Figure S1). The enthalpy of this virtual transient phase (*H*_tr_(*T*)) was calculated by first-principles electronic structure calculations and phonon mode calculation. From the relation of ${\rm{\Delta }}{H}^{E}(T)={H}_{{\rm{tr}}}(T)-({H}_{{{\rm{Fe}}}^{{\rm{II}}}{{\rm{Mn}}}^{{\rm{III}}}}(T)+{H}_{{{\rm{Fe}}}^{{\rm{III}}}{{\rm{Mn}}}^{{\rm{II}}}}(T))/2$, Δ*H* ^E^(*T*) is 7.85 kJ mol^−1^ at *T* = 325 K (Fig. 5b(i),(ii)), and the *γ* value is estimated to be 31.4 kJ mol^−1^ (=*γ*_0_). Using the *γ*_0_ value, the thermal hysteresis loop was calculated based on the SD model (Fig. 5b(iii)). Additionally, we also calculated the *x* vs*. T* curves by adopting smaller interaction parameters such as *γ*_0_/2, *γ*_0_/3, *γ*_0_/4, and *γ*_0_/10. Figure 5b(iv)–(viii) show the calculated thermal hysteresis loops for various interaction parameter values. With increasing the *γ* value, the *x* vs. *T* curve changes from a gradual continuous phase change to a first-order phase transition, and eventually to a phase transition with a thermal hysteresis loop.

For example, in the case of the *x* vs. *T* curve with *γ* (=*γ*_0_/3) = 10.5 kJ mol^−1^, the Fe^III^–Mn^II^ phase transits to Fe^II^–Mn^III^ phase at 263 K (≡ *T*_↓_: defined as the phase transition temperature in the cooling process), whereas the Fe^II^–Mn^III^ phase transits to Fe^III^–Mn^II^ phase at 364 K (≡ *T*_↑_: defined as the phase transition temperature in the warming process). The width of the thermal hysteresis loop (Δ*T* = *T*_↑_ − *T*_↓_) is 101 K. In the case of *γ* (=*γ*_0_/2) = 15.7 kJ mol^−1^, the values of *T*_↓_, *T*_↑_, and Δ*T* are 132 K, 416 K, and 284 K, respectively. Therefore, the calculation results predict that a charge-transfer induced phase transition should occur in rubidium manganese hexacyanoferrate and that a thermal hysteresis loop should appear due to a large interaction parameter value.

### Synthesis of rubidium manganese hexacyanoferrate

To confirm the prediction, we synthesized rubidium manganese hexacyanoferrate. The target compound was prepared according to a modified synthetic method from our previous report^41^. An aqueous solution of manganese(II) chloride (0.1 mol dm^−3^) was reacted with a mixed aqueous solution of potassium ferricyanide (0.1 mol dm^−3^) and rubidium chloride (1.2 mol dm^−3^). Elemental analysis shows that the formula of the obtained compound is Rb_0.94_Mn[Fe(CN)_6_]_0.98_·0.3H_2_O: Calculated; Rb, 23.06; Mn, 15.77; Fe, 15.71; C, 20.27; N, 23.64%: Found; Rb, 22.96; Mn, 15.86; Fe, 15.78; C, 20.09; N, 23.50%. The X-ray powder diffraction pattern with Rietveld analysis indicates that the crystal structure at room temperature is cubic ($F\bar{\text{4}}\text{3}m$) with a lattice constant of *a* = 10.5639(3) Å (Figure S2).

As an additional sample, Rb_0.97_Mn[Fe(CN)_6_]_0.99_∙0.3H_2_O was prepared using a different synthetic technique involving polyethylene glycol monolaurate (PGM)^46^. A PGM solution containing an aqueous solution of MnCl_2_ (0.2 mol dm^−3^) and RbCl (1 mol dm^−3^), and a PGM solution containing aqueous solution of K_3_[Fe(CN)_6_] (0.2 mol dm^−3^) and RbCl (1 mol dm^−3^) were mixed. A precipitate was obtained by centrifuging, washing in methanol, and drying in air. The X-ray powder diffraction pattern shows that the crystal structure at room temperature is cubic ($F\bar{\text{4}}\text{3}m$) with a lattice constant of *a* = 10.5606(1) Å^46^.

### IR spectra of rubidium manganese hexacyanoferrate

**Figure 6:**
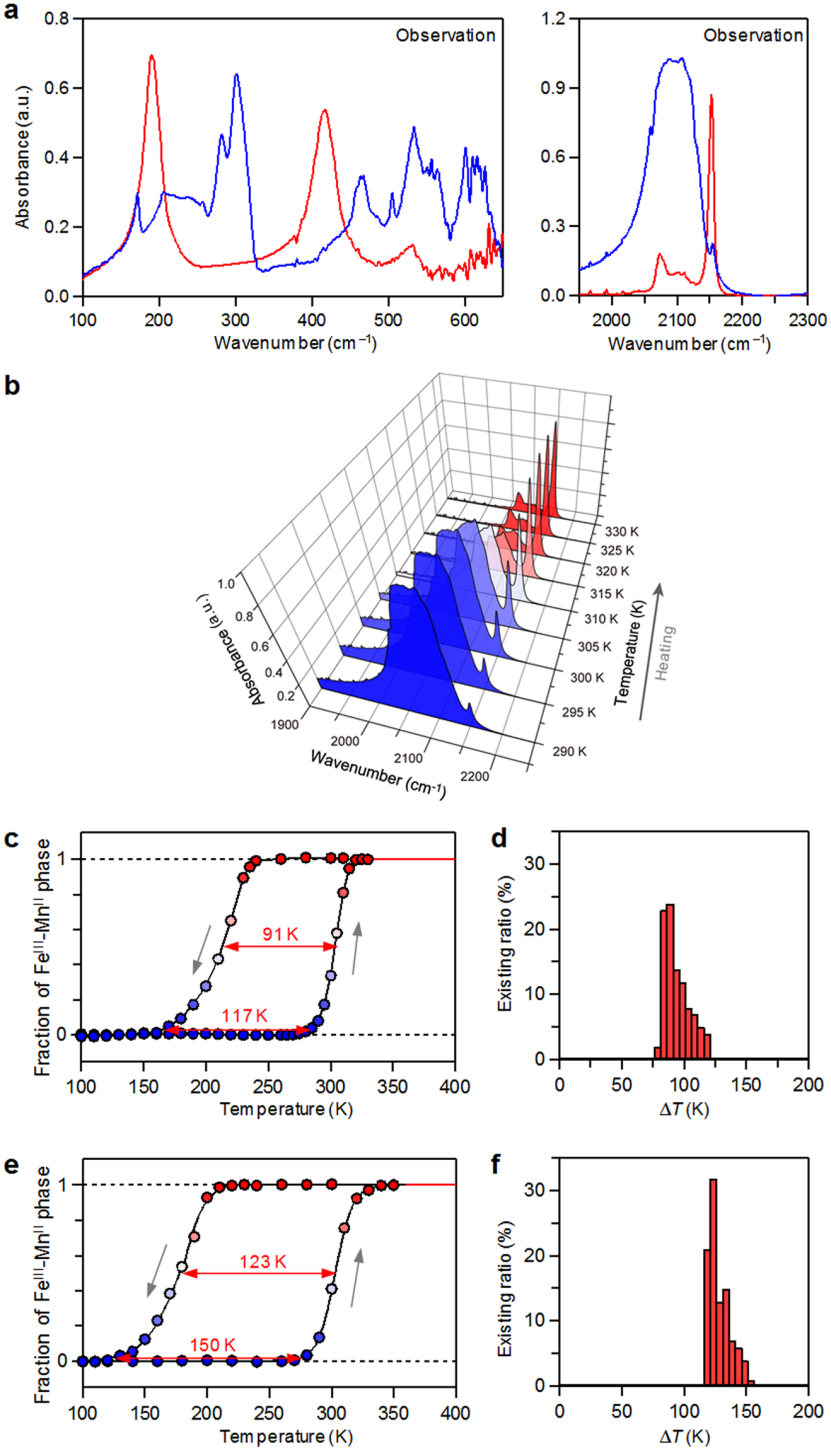
Observed far- and mid-IR spectra, and Fe^III^–Mn^II^ phase fraction vs. *T* plots of Rb_0.94_Mn[Fe(CN)_6_]_0.98_·0.3H_2_O and Rb_0.97_Mn[Fe(CN)_6_]_0.99_∙0.3H_2_O. (**a**) Observed far- and mid-IR spectra of Rb_0.94_Mn[Fe(CN)_6_]_0.98_·0.3H_2_O for the Fe^II^–Mn^III^ phase at 100 K (blue lines) and the Fe^III^–Mn^II^ phase at 300 K (red lines), respectively. (**b**) Variable temperature IR spectra of the C≡N stretching mode in the heating process for Rb_0.94_Mn[Fe(CN)_6_]_0.98_·0.3H_2_O. (**c**) Fraction of the Fe^III^–Mn^II^ phase vs. *T* plot obtained from the peak intensities of the IR spectra of Rb_0.94_Mn[Fe(CN)_6_]_0.98_·0.3H_2_O. (**d**) Histogram showing the destribution of the the thermal hysteresis width in Fig. 6c. (**e**) Fraction of the Fe^III^–Mn^II^ phase vs. *T* plot obtained from the peak intensities in the variable temperature IR spectrum of Rb_0.97_Mn[Fe(CN)_6_]_0.99_∙0.3H_2_O. (**f**) Histogram showing the destribution of the thermal hysteresis width in Fig. 6e.

The variable temperature optical phonon spectra were measured using IR spectrometry. The spectra of Rb_0.94_Mn[Fe(CN)_6_]_0.98_·0.3H_2_O are shown in Fig. 6a. Absorption peaks are observed in the IR spectra of the Fe^II^–Mn^III^ phase measured at 100 K, at 170, 230, 300, 465, 545, and 610 cm^−1^, which are assigned to the symmetric bending and asymmetric bending modes of Fe^II^–C≡N–Mn^III^ (Fig. 6a, blue lines). In the high-energy region, one broad peak is observed around 2100 cm^−1^, which is assigned to the C≡N stretching mode. On the other hand, the IR absorption spectra for the Fe^III^–Mn^II^ phase measured at 300 K are shown with red lines in Fig. 6a. The observed spectra correspond well with the calculated spectra. Therefore, the absorption peaks are assigned as follows: the peaks at 190, 415, and 530 cm^−1^ are assigned to the symmetric bending and asymmetric bending modes of Fe^III^–C≡N–Mn^II^, and the peak at 2152 cm^−1^ is assigned to the C≡N stretching mode. As for the C≡N stretching mode, the Raman activity is also confirmed by Raman spectroscopy (Figure S3).

### Observation of the phase transition and thermal hysteresis loop for Rb_0.94_Mn[Fe(CN)_6_]_0.98_·0.3H_2_O and Rb_0.97_Mn[Fe(CN)_6_]_0.99_∙0.3H_2_O

The variable temperature IR spectra and the Fe^III^–Mn^II^ phase fraction vs. *T* plots of Rb_0.94_Mn[Fe(CN)_6_]_0.98_·0.3H_2_O are shown in Fig. 6b and c, respectively. By increasing *T* at a heating rate of +0.5 K min^−1^, the Fe^III^–Mn^II^ phase appears at 304 K (≡*T*_↑,obs_: defined as the temperature with 50% of Fe^III^–Mn^II^ phase in the warming process). With decreasing *T*, the Fe^II^–Mn^III^ phase appears at 213 K (≡*T*_↓,obs_: defined as the temperature with 50% fraction in the cooling process). The phase transition temperature, defined as *T*_p,obs_ = (*T*_↑,obs_ + *T*_↓,obs_)/2, is 259 K. This *T*_p,obs_ value is 80% of the predicted phase transition temperature of *T*_p_ = 325 K mentioned above. The width of the thermal hysteresis loop, defined as ∆*T*_obs_ = *T*_↑,obs_−*T*_↓,obs_, of this system is 91 K. It is noted that, the width of the thermal hysteresis loop Δ*T* is not a unique value but is spread in the range of 79 K ≤ Δ*T* ≤ 117 K as shown in Fig. 6d. Such a broadness of Δ*T* corresponds to the width of the calculated hysteresis loop with *γ* in the range of 9.7 kJ mol^−1^ ≤ *γ* ≤ 10.4 kJ mol^−1^.

Rb_0.97_Mn[Fe(CN)_6_]_0.99_∙0.3H_2_O shows a phase transition with *T*_↑,obs_ = 301 K, *T*_↓,obs_ = 178 K, and then *T*_p,obs_ = 240 K. The hysteresis width ∆*T*_obs_ is a large value of 123 K (Fig. 6e). This ∆*T*_obs_ value is close to the calculated ∆*T* value with *γ* = 11.3 kJ mol^−1^ (=*γ*_0_/2.8). Additionally, in the thermal hysteresis, components of ∆*T* approaching 150 K are also included, i.e., 117 K ≤ Δ*T* ≤ 150 K (Fig. 6f), which corresponds to 11.0 kJ mol^−1^ ≤ *γ* ≤ 12.2 kJ mol^−1^. The results of these two samples show that the *γ* value is in the range of *γ*_0_/3 < *γ* < *γ*_0_/2, indicating that the theoretical prediction is reasonable.

Let us consider *γ* in the present system. It is known that *γ* can be expressed as *γ* = 2 *γ*_αβ_ − *γ*_αα_ − *γ*_ββ_, where *γ*_αα_ is the interaction parameter between α-sites, *γ*_αβ_ is the interaction parameter between α- and β-sites, and *γ*_ββ_ is the interaction parameter between β-sites^47^. The following effects are known as the origins of the interaction at the atomic level: electrostatic interaction between coordinated molecules^48^, intermolecular coupling of the molecular distortions^49–51^, elastic interaction between metal ions of different spins^52–54^, and electron–phonon coupling^55^. In the light of these reports, the origin of *γ* in the present charge-transfer phase transition system is considered as electrostatic interaction among charge-transferred sites (Mn^III/II^ and Fe^II/III^), Jahn-Teller distortion on Mn^III^, and elastic interaction between metal ions of different spins.

The magnitude of the *γ* value depends on the structure of the assumed virtual transient phase. Although there are some differences between the predictions and the experimental results, the calculations agree with the observations fairly well, indicating that a prediction of a phase transition is possible using the present strategy. As an additional note, there are defects in the real systems of Rb_0.94_Mn[Fe(CN)_6_]_0.98_·0.3H_2_O and Rb_0.97_Mn[Fe(CN)_6_]_0.99_∙0.3H_2_O, which are considered to weaken the interaction parameter compared to the ideal crystal structure of RbMn[Fe(CN)_6_] (Figure S4). Furthermore, such defects in the crystal have a possibility of causing domain nucleation and accelerating domain growth^56^. In this case, the width of thermal hysteresis loop would become narrower compared to the ideal crystal system.

## Conclusion

In this study, we proposed a logical strategy to design a phase transition material accompanying a thermal hysteresis loop. The strategy is as follows: (i) The thermodynamic parameters of enthalpy, entropy, and Gibbs energy are calculated by first-principles electronic structure calculations and phonon mode calculations, and the possibility of a phase transition and the transition temperature are evaluated. (ii) Then, whether the system has a thermal hysteresis or not and the width of the thermal hysteresis loop are determined using a statistical thermodynamic mean-field theory.

As a demonstration, we investigated the charge-transfer phase transition on a rubidium manganese hexacyanoferrate. The predicted phase transition temperature and the thermal hysteresis loop agree well with the experimental results. The approach shown in this paper could be applied to structural phase transitions such as charge-transfer, spin-transition, metal-insulator phase transitions, etc., and will contribute to the rapid development of yet undiscovered phase transition materials. Moreover, if the flowchart for a logical strategy to predict the phase transition shown in this study is eventually implemented to artificial intelligence (AI), numerous phase transition materials could be discovered inside a computer.

## Methods

### Flowchart of the strategy for theoretical prediction

The formation enthalpies at zero kelvin of the α- and β-phases, *H*_F,α_ and *H*_F,β_, are obtained by first-principles periodic structure calculations of the electronic structures (Fig. 1, upper). The temperature dependence of the enthalpies due to the lattice vibrations can be obtained by first-principles phonon mode calculations, *H*_vib,α_(*T*) and *H*_vib,β_(*T*). The sum of *H*_F,α_ and *H*_vib,α_(*T*) shows the temperature dependence of enthalpy, *H*_α_(*T*). In a similar manner, *H*_β_(*T*) is obtained. The phonon mode calculations also provide the vibrational entropies of the α- and β-phases, *S*_vib,α_(*T*) and *S*_vib,β_(*T*), respectively. Additionally, the degeneracy of the orbitals and the multiplicity of the spins contribute to the entropy as orbital-and-spin entropy, *S*_os_. The *S*_os_ value is obtained by *R*ln*W*, where *R* is the gas constant and *W* = (orbital degeneracy) × (spin multiplicity) = (2*l* + 1)(2*s* + 1). Therefore, *S*(*T*) is expressed as the sum of *S*_vib_(*T*) and *S*_os_ (i.e., *S*_α_(*T*) = *S*_vib,α_(*T*) + *S*_os,α_ and *S*_β_(*T*) = *S*_vib,β_(*T*) + *S*_os,β_). Based on the aforementioned thermodynamic parameters, the Gibbs energies for the α- and β-phases, *G*_α_(*T*) and *G*_β_(*T*), are evaluated. The crossover temperature between *G*_α_(*T*) and *G*_β_(*T*) corresponding to the phase transition temperature, *T*_p_, is predicted.

To determine whether a system has a thermal hysteresis, the SD model was adopted (Fig. 1, lower). In the SD model, the excess enthalpy Δ*H* ^E^ is expressed by *γx*(1 − *x*), where *γ* is the interaction parameter due to the interaction between the A–B and A^+^–B^−^ units, and *x* is the fraction of the A^+^–B^−^ unit. To evaluate *γ*, we need to consider the virtual transient phase (tr-phase), such as the –A–B–A^+^–B^–^ phase. The enthalpy of this virtual transient phase *H*_tr_(*T*) was calculated by first-principles electronic structure calculations and phonon mode calculations.

The Δ*H* ^E^(*T*) value corresponds to the difference between *H*_tr_(*T*) and the average formation enthalpy of the α- and β-phases (i.e., Δ*H* ^E^(*T*) = *H*_tr_(*T*)−{*H*_α_(*T*) + *H*_β_(*T*)}/2). From the relation of Δ*H* ^E^(*T*) = *γx*(1 − *x*), *γ* is obtained. Then the *x* vs. *T* plots can be evaluated by the SD model. The Gibbs energy in the SD model, *G*_SD_(*T*), is described by *G*_SD_(*T*) = *x*(*H*_β_(*T*) − *H*_α_(*T*)) + *γx*(1 − *x*) + *T*{*R*[*x*ln*x* + (1 − *x*)ln(1 − *x*) − *x*(*S*_β_(*T*) − *S*_α_(*T*))]}. From the results of the SD model calculations, whether a thermal hysteresis loop appears or not can be determined.

### First-principles calculations of formation enthalpies

The formation enthalpies of the Fe^II^–Mn^III^ and Fe^III^–Mn^II^ phases and the virtual transient phase were calculated by first-principles calculations using VASP, which is a plane-wave projector augmented wave (PAW) method program^57,58^. To obtain accurate values, HSE06 hybrid functional was used for the exchange-correlation energy^59–62^. The range separation parameter of 0.2 Å^−1^ was adopted for HSE06. The plane-wave cutoff energy was set to 500 eV, and the electronic iterations convergence were 1 × 10^−6^ eV. Calculations were performed with *k*-mesh of 3 × 3 × 3 for all phases.

### First-principles calculations of phonon modes

First-principles phonon mode calculations based on density functional theory were conducted for rubidium manganese hexacyanoferrate, RbMn[Fe(CN)_6_], using the Phonon code by GGA + U/PBE. Wave functions based on the plane waves and the potentials of the core orbitals were represented by the projector-augmented wave of Blöchl. The exchange-correlation term was evaluated by the generalized gradient approximation by Perdew, Burke, and Ernzerhof. The reported tetragonal and cubic lattice parameters for the Fe^II^–Mn^III^ and Fe^III^–Mn^II^ phases of rubidium manganese hexacyanoferrate were used as the initial structures in the computed models^41^. The lattice parameters and atomic positions were optimized with an energy cutoff of 500 eV and a 3 × 3 × 3 *k-*mesh until satisfying a 10^−5^ eV pm^−1^ force tolerance. √2 × √2 × 1 supercells of the optimized structures were used to calculate the phonon modes of RbMn[Fe(CN)_6_], which were calculated by the direct method implemented in Phonon code with 2-pm displacements using the optimized atomic positions. *U* − *J* value was set to 4.0 eV for Fe and Mn (Supplementary Information).

### Measurements

Elemental analyses for Rb, Mn, and Fe were performed by HP4500 inductively coupled plasma mass spectroscopy, while those for C and N were performed by standard microanalytical methods. X-ray powder diffraction measurements were performed with a Rigaku Ultima IV diffractometer with Cu *K*_*α*_ radiation (*λ* = 1.5418 Å). The PDXL program (Rigaku) was used for the Rietveld analyses. For the spectroscopic measurements, a JASCO 6100 spectrometer was used in the energy region of 100–650 cm^−1^, and a Shimadzu FT-IR 8200PC spectrometer was used in the energy region of 1950–2300 cm^−1^. Raman spectra were measured with a Raman microspectrometer (JASCO NRS-5100).

## Electronic supplementary material

Supplementary Information

Supplementary Movie S1

Supplementary Movie S2
