# Supplementary material for: Theoretical prediction of a charge-transfer phase transition
Source: Sci Rep. 2018 Jan 11;8:63. doi: 10.1038/s41598-017-18213-0 (PMC5765035; doi:10.1038/s41598-017-18213-0)
Supplement: Supplementary file 1 — Supplementary Information [file 41598_2017_18213_MOESM1_ESM.pdf]

Theoretical prediction of charge-transfer phase transition

Hiroko Tokoro,[1,2,*] Asuka Namai,[1] Marie Yoshikiyo,[1] Rei Fujiwara,[2] Kouji Chiba,[3] and Shin-ichi Ohkoshi,[1,4,*]

[1] *Department of Chemistry, School of Science, The University of Tokyo, 7-3-1 Hongo, Bunkyo-ku, Tokyo 113-0033, Japan*
[2] *Division of Materials Science, Faculty of Pure and Applied Sciences, University of Tsukuba, 1-1-1 Tennodai, Tsukuba, Ibaraki 305-8573, Japan*
[3] *Material Science Div., MOLSIS Inc., Tokyo Daia Bldg., 1-28-38 Arakawa, Chuo-ku, Tokyo 104-0033, Japan*
[4] *Cryogenic Research Center, The University of Tokyo, 2-11-16 Yayoi, Bunkyo-ku, Tokyo 113-0032, Japan*

Contents

			Page
§ 1.	**Legends for Supplementary Movies S1 and S2.**		**S2**
§ 2.	**Crystal structure and calculated phonon frequencies of the Fe^{II}–Mn^{III} phase.**	Table S1	**S3**
§ 3.	**Crystal structure and calculated phonon frequencies of the Fe^{III}–Mn^{II} phase.**	Table S2	**S4**
§ 4.	**First-principles phonon mode calculations.**		**S5**
§ 5.	**Contribution from orbital degeneracy and spin multiplicity on the entropy.**		**S5**
§ 6.	**Schematic crystal structure of the virtual transient structure.**	Fig. S1	**S6**
§ 7.	**Crystal structure analysis of the synthesized rubidium manganese hexacyanoferrate.**	Fig. S2	**S7**
§ 8.	**Raman spectra of $Rb_{0.94}Mn[Fe(CN)_6]_{0.98}\cdot 0.3H_2O$.**	Fig. S3	**S8**
§ 9.	**Defect in the experimentally obtained rubidium manganese hexacyanoferrate.**	Fig. S4	**S9**

§ 1. Legends for Supplementary Movies S1 and S2.

Supplementary Movie S1: Calculated phonon modes at 302.4 cm^{-1}, 525.0 cm^{-1}, and 2130 cm^{-1} for the Fe^{II}–Mn^{III} phase in $RbMn[Fe(CN)_6]$. The first part of the movie shows the atomic movement of the phonon mode at 302.4 cm^{-1}, and the second part of the movie shows the atomic movement of the phonon mode at 525.0 cm^{-1}. These phonon modes are due to the symmetric bending and asymmetric bending modes of Fe–C≡N–Mn. The last part of the movie shows the atomic movement of the phonon mode at 2130 cm^{-1} assigned to the stretching mode of C≡N. Green, blue, and light blue spheres represent Rb, Mn, and Fe atoms, respectively, and gray spheres represent C and N atoms.

Supplementary Movie S2: Calculated phonon mode at 189.2 cm^{-1}, 411.2 cm^{-1}, and 2179 cm^{-1} for the Fe^{III}–Mn^{II} phase in $RbMn[Fe(CN)_6]$. The first part of the movie shows the atomic movement of the phonon mode at 189.2 cm^{-1}, and the second part of the movie shows the atomic movement of the phonon mode at 411.2 cm^{-1}. These phonon modes are due to the symmetric bending and asymmetric bending modes of Fe–C≡N–Mn. The last part of the movie shows the atomic movement of the phonon mode at 2179 cm^{-1} assigned to the stretching mode of C≡N. Green, red, and yellow spheres represent Rb, Mn, and Fe atoms, respectively, and gray spheres represent C and N atoms.

§ 2. Crystal structure and calculated phonon frequencies of the Fe^{II}–Mn^{III} phase.

Table S1. Crystal structure (upper) and calculated phonon frequencies obtained by first-principles calculations (lower) of the Fe^{II}–Mn^{III} phase. The list contains the optical phonon frequencies at the Brillouin zone center, Γ points, multiplicities, irreducible representations, and IR and Raman activities.

Crystal system		Tetragonal		
Space group		$I\bar{4}m2$		
a (Å)		7.090		
b (Å)		7.090		
c (Å)		10.520		
Atoms	*x*	*y*	*z*	Occupancy
Rb(1)	0.0000	0.50000	0.2500	1
Mn(1)	0.0000	0.0000	0.5000	1
Fe(1)	0.0000	0.0000	0.0000	1
C(1)	0.0000	0.0000	0.1738	1
C(2)	0.1882	0.1882	0.0000	1
N(1)	0.0000	0.0000	0.2847	1
N(2)	0.3014	0.3014	0.0000	1

No.	Frequency (cm^{-1})	Multiplicity	Irreducible representations	Activity	
				IR	Raman
1	39.3	1	B_2	O	O
2	63.8	2	E	O	O
3	67.2	1	A_2	–	–
4	144.3	1	B_2	O	O
5	159.4	2	E	O	O
6	186.8	1	A_2	–	–
7	226.0	2	E	O	O
8	258.0	2	E	O	O
9	278.0	1	B_2	O	O
10	289.7	1	B_2	O	O
11	296.4	2	E	O	O
12	354.6	1	A_2	–	–
13	362.3	2	E	O	O
14	457.6	1	B_2	O	O
15	465.0	1	A_1	–	O
16	471.5	1	A_2	–	–
17	476.0	2	E	O	O
18	506.5	2	E	O	O
19	508.8	1	B_2	O	O
20	528.4	2	E	O	O
21	531.8	1	B_1	–	O
22	575.7	1	A_1	–	O
23	603.1	1	B_2	O	O
24	630.4	2	E	O	O
25	2078.6	2	E	O	O
26	2085.0	1	B_1	–	O
27	2110.2	1	B_2	O	O
28	2116.3	1	A_1	–	O
29	2182.7	1	A_1	–	O

§ 3. Crystal structure and calculated phonon frequencies of the Fe^{III}–Mn^{II} phase.

Table S2. Crystal structure (upper) and calculated phonon frequencies obtained by first-principles calculations (lower) of the Fe^{III}–Mn^{II} phase. The list contains the optical phonon frequencies at the Brillouin zone center, Γ points, multiplicities, irreducible representations, and IR and Raman activities.

Crystal system	Cubic
Space group	$F\bar{4}3m$
a (Å)	10.533
b (Å)	10.533
c (Å)	10.533

Atoms	*x*	*y*	*z*	Occupancy
Rb(1)	0.2500	0.2500	0.2500	1
Mn(1)	0.0000	0.0000	0.0000	1
Fe(1)	0.5000	0.5000	0.5000	1
C(1)	0.0000	0.0000	0.3050	1
N(1)	0.0000	0.0000	0.2068	1

No.	Frequency (cm^{-1})	Multiplicity	Irreducible representations	Activity	
				IR	Raman
1	-40.0	3	T_2	O	O
2	119.8	3	T_1	–	–
3	194.6	3	T_2	O	O
4	204.6	3	T_1	–	–
5	269.9	3	T_2	O	O
6	288.7	3	T_2	O	O
7	363.8	2	E	–	O
8	367.7	1	A_1	–	O
9	379.0	3	T_1	–	–
10	394.4	3	T_2	O	O
11	476.0	3	T_1	–	–
12	494.7	3	T_2	O	O
13	524.3	3	T_2	O	O
14	2025.9	3	T_2	O	O
15	2028.9	2	E	–	O
16	2079.1	1	A_1	–	O

§ 4. First-principles phonon mode calculations..

The reason why $U - J = 4$eV in GGA+U is used for the phonon mode calculation is as follows. We have to use the same $U - J$ value for Fe^{II}–Mn^{III} and Fe^{III}–Mn^{II} phases. The value of $(U - J)$/eV should be the number of *d*-electrons on the metal ions or a smaller number. For example, in Fe^{III} ($3d^5$)–CN –Mn^{II} ($3d^5$) phase, the numbers of the *d*-electrons are 5, and thus, $U - J$ value should be below 5 eV. In Fe^{II} ($3d^6$)–CN–Mn^{III} ($3d^4$) phase, the numbers of *d*-electrons are 6 and 4 for Fe^{II} and Mn^{III}, respectively, and hence, $U - J$ should be below 4 eV, *i.e.*, $U - J \leq 4$eV. Thus, we used $U - J = 4$ eV as the common parameter for the Fe^{II}–Mn^{III} and Fe^{III}–Mn^{II} phases. It is noted that we also investigated a calculation with $U - J = 0$ eV. The result with $U - J = 0$ eV shows a disappearance of the band gap, i.e., conducting property, despite the isolating property of the present system. A small $U - J$ value is not appropriate for the present system, and we used $U - J =$ 4 eV as the maximum value in the condition of $U - J \leq 4$eV.

§ 5. Contribution from orbital degeneracy and spin multiplicity on the entropy.

Contributions from the orbital degeneracy and the spin multiplicity, i.e., S_{os} values, are Rln5 for the Fe^{II}–Mn^{III} phase and Rln36 for the Fe^{III}–Mn^{II} phase. This is because the Fe^{II}–Mn^{III} phase consists of $Fe^{II}(^1A_{1g})$ and $Mn^{III}(^5B_{1g})$, in which $^1A_{1g}$ has a one (= 1 (orbital degeneracy) $\times$ 1 (spin multiplicity))-fold degeneracy and $^5B_{1g}$ has a five (= 1×5)-fold degeneracy. Therefore, the degeneracy of the Fe^{II}–Mn^{III} phase is five (= 1×5)-fold. In contrast, the degeneracy of the Fe^{III}–Mn^{II} phase is 36 (= 6×6)-fold since the Fe^{III}–Mn^{II} phase consists of $Fe^{III}(^2T_{2g})$ and $Mn^{II}(^6A_{1g})$, where $^2T_{2g}$ has a six (= 3×2)-fold degeneracy and $^6A_{1g}$ has a six (= 1×6)-fold degeneracy.

§ 6. Schematic crystal structure of the virtual transient structure.

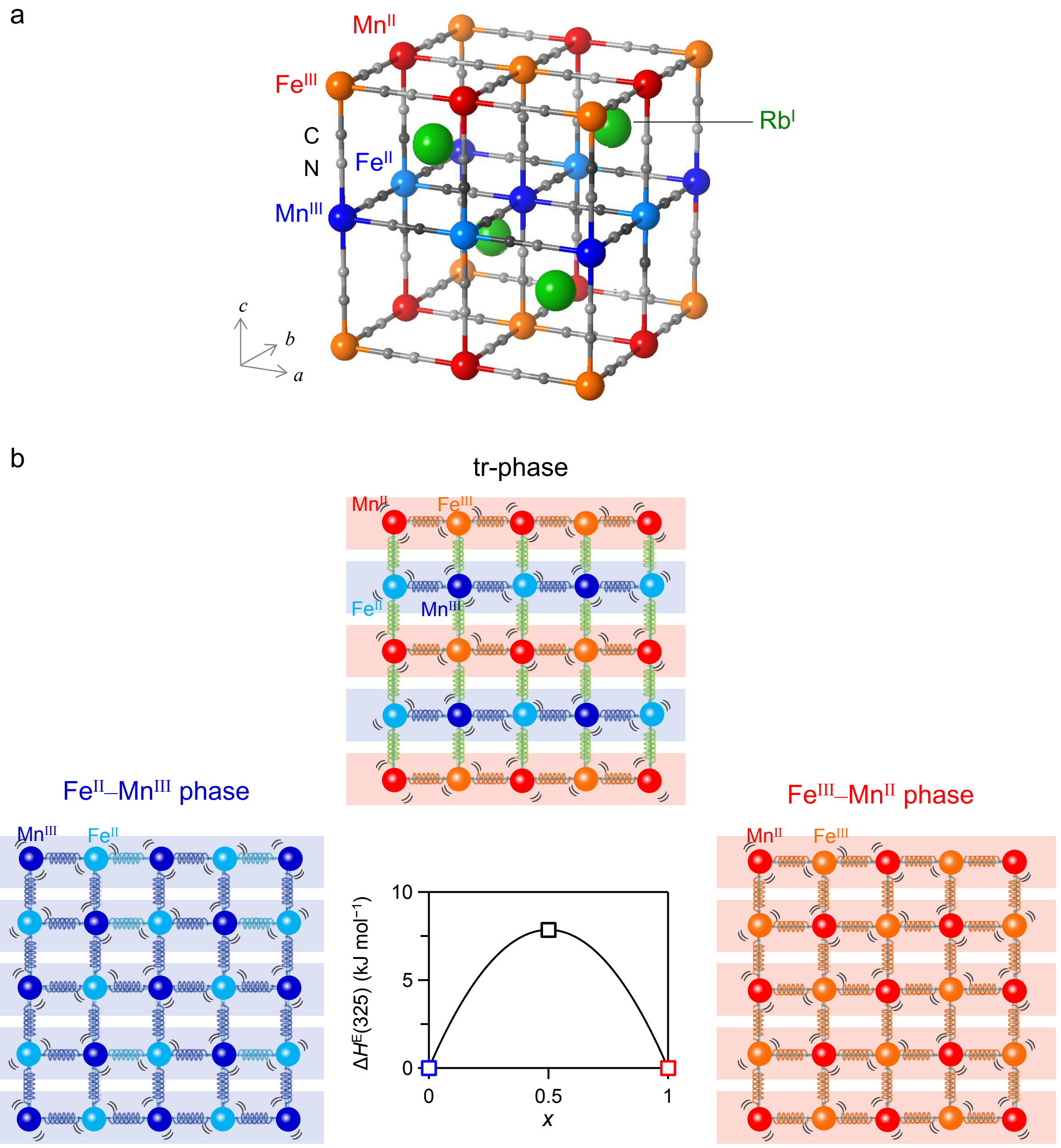

Figure S1. a, Schematic crystal structure of the virtual transient structure as an alternating mixed phase of (A–B layer)-by-(A^{+}–B^{-} layer) (i.e., (Fe^{II}–Mn^{III} layer)-by-(Fe^{III}–Mn^{II} layer)) along the *c*-axis of the tetragonal structure ($a = b \neq c$). Green, blue, light blue, red, and orange spheres represent Rb^{I}, Mn^{II}, Fe^{II}, Mn^{II}, and Fe^{III}, respectively. **b**, Schematic illustration of Fe^{II}–Mn^{III} structure (lower left), layer-by-layer structure (tr-phase) (upper middle), and Fe^{III}–Mn^{II} structure (lower right). Diagram in the center shows the calculated excess enthalpies at the transition temperature ($\Delta H^{E}(325)$) for the Fe^{II}–Mn^{III} phase (blue square), tr-phase (black square), and Fe^{III}–Mn^{II} phase (red square).

§ 7. Crystal structure analysis of the synthesized rubidium manganese hexacyanoferrate.

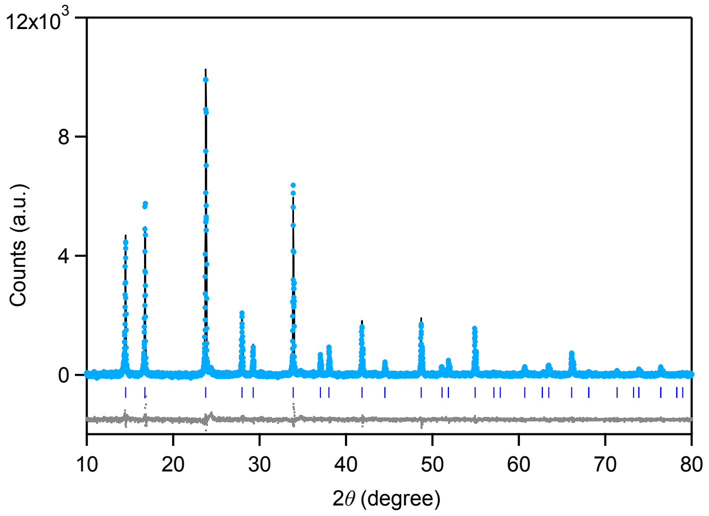

Figure S2. XRD pattern and Rietveld analysis of the synthesized rubidium manganese hexacyanoferrate, $Rb_{0.94}Mn[Fe(CN)_6]_{0.98}\cdot 0.3H_2O$, at room temperature. Light blue dots, black line, and gray dots are the observed pattern, calculated pattern, and their difference, respectively. Blue bars represent the calculated positions of the Bragg reflections of the cubic structure ($F\bar{4}3m$), with a lattice constant of a=10.5639(3) Å.

§ 8. Raman spectra of $Rb_{0.94}Mn[Fe(CN)_6]_{0.98}·0.3H_2O$.

We performed Raman spectroscopy measurements. The Raman spectrum of the Fe^{III}–Mn^{II} phase shows a strong peak at 2162 cm^{-1} with a shoulder peak at 2155 cm^{-1}. These peaks almost correspond to the calculated Raman active modes at 2220 cm^{-1} and 2212 cm^{-1}. On the other hand, in the Raman spectrum of the Fe^{II}–Mn^{III} phase, peaks are observed at 2091 cm^{-1}, 2109 cm^{-1}, 2162 cm^{-1}, and 2202 cm^{-1}. The peaks at 2091 cm^{-1}, 2109 cm^{-1}, and 2202 cm^{-1} correspond to the calculated peaks due to the C≡N stretching modes at 2127 cm^{-1}, 2148 cm^{-1}, and 2207 cm^{-1}. The peak at 2162 cm^{-1} is due to the remaining Fe^{III}–Mn^{II} phase. The Fe^{III}–Mn^{II} phase was remained because the measurement was conducted at room temperature after cooling the sample by indirect contact with liquid N_2.

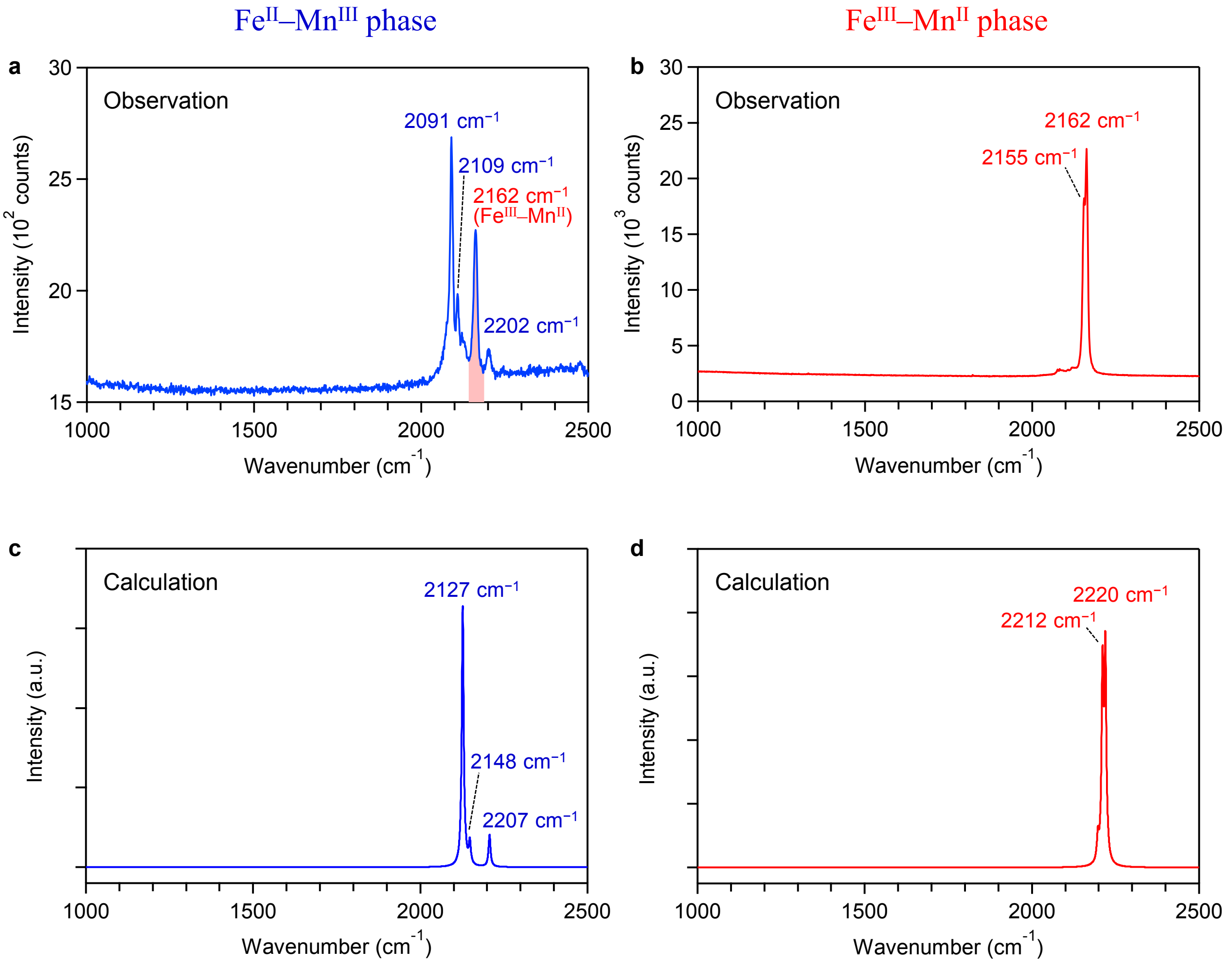

Figure S3. Raman spectra of $Rb_{0.94}Mn[Fe(CN)_6]_{0.98}·0.3H_2O$. **a**, Observed spectrum of the Fe^{II}–Mn^{III} phase measured at room temperature after cooling the sample by indirect contact with liquid N_2. Pink shadow indicates the peak assigned to the remaining Fe^{III}–Mn^{II} phase. **b**, Observed spectrum of the Fe^{III}–Mn^{II} phase measured at room temperature. **c**, Calculated Raman spectrum using the probability of each phonon mode of the Fe^{II}–Mn^{III} phase. **d**, Calculated Raman spectrum using the probability of each phonon mode of the Fe^{III}–Mn^{II} phase.

§ 9. Defect in the experimentally obtained rubidium manganese hexacyanoferrate.

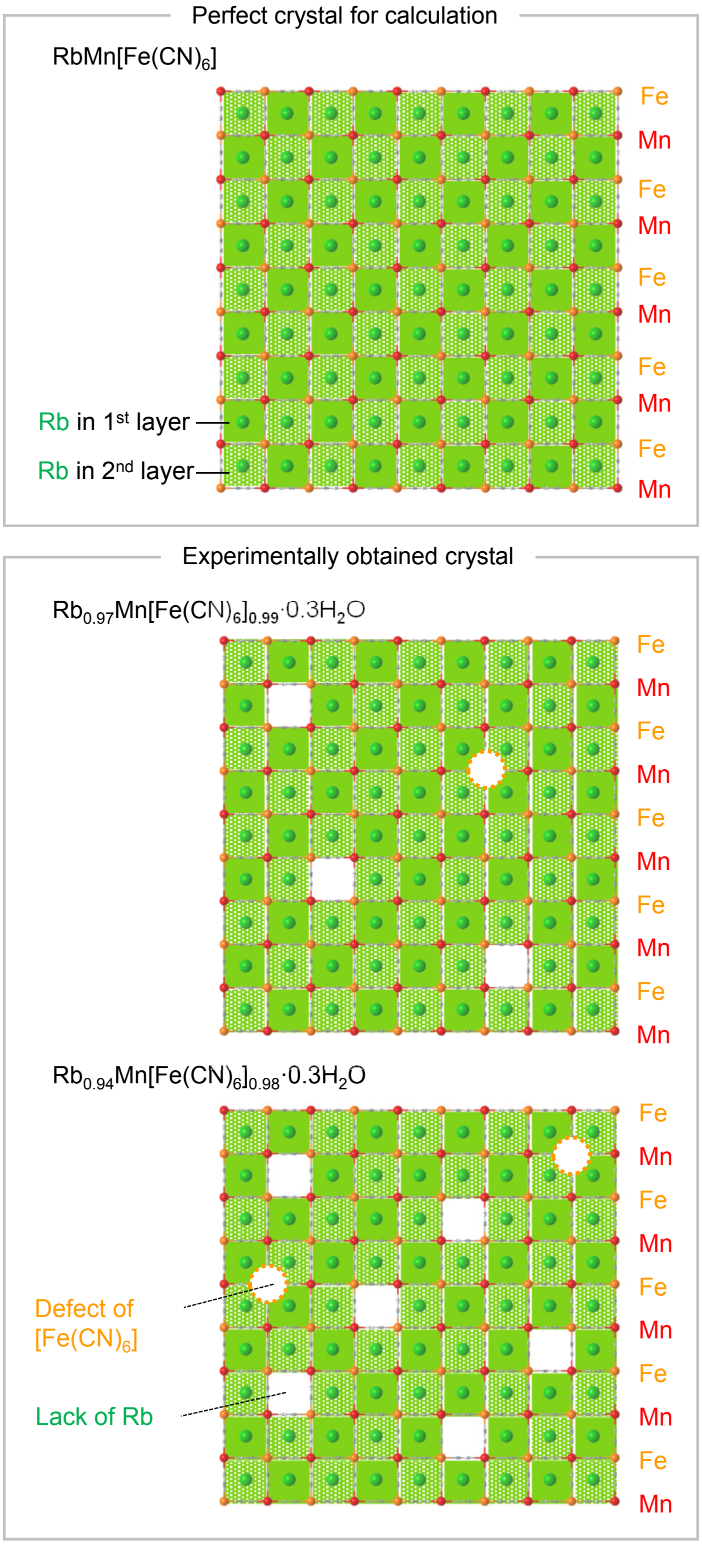

Figure S4. Schematic illustration of the crystal lattice for a perfect crystal for model calculation ($RbMn[Fe(CN)_6]$) (upper) and experimentally obtained crystals ($Rb_{0.97}Mn[Fe(CN)_6]_{0.99}\cdot 0.3H_2O$ and $Rb_{0.94}Mn[Fe(CN)_6]_{0.98}\cdot 0.3H_2O$) (lower).
